# Stereotactic body radiotherapy in patients with bone oligometastases from breast cancer – results from a European multicenter cohort study

**DOI:** 10.1016/j.ctro.2026.101142

**Published:** 2026-03-03

**Authors:** Sebastian Schäfer, Isabell Seiler, Lena Kästner, Johannes Meents, Marek Slavik, Petr Burkon, Mauro Loi, Pierluigi Bonomo, Camilla von Wachter, Panagiotis Balermpas, Anna Sabrina Schunn, Sophia Drabke, Richard Holy, Kenneth Klischies, Olaf Wittenstein, Jochen Willner, Andrea Baehr, Priska Bank, Richard Partl, Thomas Mader, Bernd Frerker, Fabian Lohaus, Felix Ehret, Maike Trommer, Eleni Gkika, Alexander Rühle, Matthias Guckenberger, Christos Moustakis, Thomas Brunner, Oliver Blanck, Judit Boda- Heggemann, Nils H. Nicolay, Franziska Nägler

**Affiliations:** a Department of Radiotherapy, University Hospital Leipzig, Stephanstraße 9a, Leipzig, Germany; b Comprehensive Cancer Center Central Germany, Partner Site Leipzig, Leipzig, Germany; c Department of Radiation Oncology, University Medicine Mannheim, Medical Faculty Mannheim, Heidelberg University, Theodor-Kutzer-Ufer 1-3, Mannheim, Germany; d DKFZ Hector Cancer Institute at the University Medical Center Mannheim, Mannheim, Germany; e Department of Radiation Oncology, Masaryk Memorial Cancer Institute and Medical Faculty, Masaryk University, Brno, Czech Republic; f Radiation Oncology Unit, Azienda Ospedaliero-Universitaria Careggi, University of Florence, Florence, Italy; g Department of Radiation Oncology, University Hospital Zurich, University of Zurich, Rämistr. 100, Zurich CH-8091, Switzerland; h Department of Radiotherapy and Radiation Oncology, University Medical Center Mainz, Langenbeckstraße 1, 55131 Mainz, Germany; i Radiotherapy Med 360 Grad in Aachen, Viehhofstraße 43, 52066 Aachen, Germany; j Department of Radiation Oncology, University Medical Center Schleswig-Holstein, Kiel, Germany; k Department of Radiotherapy, Klinikum Bayreuth GmbH, Preuschwitzer Str. 101, 95445 Bayreuth, Germany; l Department of Radiation Oncology, University Medical Center Hamburg-Eppendorf, Hamburg, Germany; m Clinic for Radiation Therapy and Radio-Oncology Gera, Waldklinikum Gera, Germany; n Department of Therapeutic Radiology and Oncology, Comprehensive Cancer Center, Medical University of Graz, 8036 Graz, Austria; o Department of Radiation Oncology, Kantonsspital Graubünden, Chur, Switzerland; p Department of Radiation Oncology, Rostock University Medical Center, Rostock, Germany; q Department of Radiotherapy and Radiation Oncology, Faculty of Medicine and University Hospital Carl Gustav Carus, TUD Dresden University of Technology, Fetscherstraße 74, Dresden, Germany; r National Center for Tumor Diseases (NCT), NCT/UCC Dresden, a Partnership between DKFZ, Faculty of Medicine and University Hospital Carl Gustav Carus, TUD Dresden University of Technology, and Helmholtz-Zentrum Dresden-Rossendorf (HZDR), Germany; s OncoRay – National Center for Radiation Research in Oncology, Faculty of Medicine and University Hospital Carl Gustav Carus, TUD Dresden University of Technology, Helmholtz-Zentrum Dresden-Rossendorf, Dresden, Germany; t German Cancer Consortium (DKTK), Partner Site Dresden, and German Cancer Research Center (DKFZ), Heidelberg, Germany; u European Radiosurgery Center Munich, Munich, Germany; v Charité – Universitätsmedizin Berlin, Corporate Member of Freie Universität Berlin and Humboldt-Universität zu Berlin, Department of Radiation Oncology, Berlin, Germany; w German Cancer Consortium (DKTK), Partner Site Berlin, a Partnership between DKFZ and Charité – Universitätsmedizin Berlin, Germany; x Department of Radiation Oncology, Faculty of Medicine and University Hospital Bonn, Bonn, Germany; y Center for Integrated Oncology CIO Aachen Bonn Cologne Duesseldorf, Department of Internal Medicine, Faculty of Medicine and University Hospital Cologne, Germany

**Keywords:** Bone metastases, Breast cancer, Stereotactic radiotherapy, Stereotactic body radiotherapy, SBRT, Metastasis-directed therapy (MDT), Spine metastases, Dose concept

## Abstract

**Background and purpose:**

The interdisciplinary treatment concept for bone metastases (BoM) of oligometastatic breast cancer includes metastasis-directed radiotherapy. We evaluated oncological outcomes of BoM treated with stereotactic body radiotherapy (SBRT) in a large European cohort.

**Material and methods:**

Data of breast cancer patients treated with SBRT for BoM between 2010 and 2024 at 17 European cancer centers were retrospectively collected. Treatment and dose concepts were analyzed regarding their impact on freedom from local recurrence (FFLR), overall survival (OS), progression-free survival (PFS), and safety profile.

**Results:**

With a median follow-up of 29.6 months (interquartile range: 15.0–48.0), 109 patients with 147 BoM were analyzed. SBRT was performed with a prescribed dose of median 35 Gy in median 5 fractions. 1-/3-year FFLR for mean biologically effective dose (BED_10_) ≥ 50 Gy in the gross tumor volume (GTV_mean_) was 98.1% (95%-CI: 92.7%-99.5%) and 93.9% (95%-CI: 85.5%-97.5%), respectively. For GTV_mean_ BED_10_ ≥ 50 Gy, 1-/3-year PFS was 62.1% (95%-CI: 43.6%-76.0%)/26.7% (95%-CI: 11.0%-45.4%) for non-spine BoM and 66.2% (95%-CI: 50.8%-77.8%)/35.8% (95%-CI: 21.3%-50.4%) for spine BoM. In multivariable analysis, systemic therapy (HR [hazard ratio] 0.22; p < 0.001) was associated with improved OS, while higher GTV_mean_ BED_10_ (HR 0.96; p < 0.001) was associated with improved PFS and spine localization with worse PFS (HR 1.81; p = 0.02). Adverse events were rare, with fracture rates of 2.7%.

**Conclusion:**

In this multicenter retrospective cohort analysis, SBRT of BoM from breast cancer was a well-tolerated, effective treatment. Prospective studies are needed to investigate these findings further and determine the role of standardized SBRT concepts in the multidisciplinary management of oligometastatic breast cancer.

## Highlights

• Stereotactic radiotherapy of bone oligometastases with excellent local control.
• Well-tolerated treatment with especially low fracture rates.
• Prospective studies are needed to determine the role of standardized SBRT concepts.

## Introduction

In global cancer statistics for 2022, cancer of the female breast was the second most common cancer diagnosis worldwide, accounting for 11.6% of all cancers globally [1]. It is known that up to 75% of patients with advanced breast cancer (BC) present with bone metastases (BoM), which, depending on the subtype, occur more frequently in inflammatory BC than in lobular or infiltrating ductal tumors [2], [3]. BoM are a major cause of skeletal-related events (SREs) such as pain, pathological fractures, hypercalcemia, and neurological impairment [4], [5]. The addition of metastasis-directed therapy (MDT), such as guideline-recommended radiotherapy, to the existing broad range of systemic treatment options can prevent these SREs and maintain quality of life [6], [7]. In a recently reported randomized phase-II trial, even the prophylactic radiation therapy for asymptomatic, high-risk BoM, including a high proportion of BC patients, reduced SREs and hospitalizations [8]. Recent clinical studies on stereotactic body radiotherapy (SBRT) also revealed clinical benefits in terms of local control and oncological outcome, and in selected cases, SBRT has demonstrated effectiveness in treating oligometastatic disease, leading to the development of practice guidelines for bone SBRT [9], [10], [11], [12], [13].

However, the results of clinical studies on SBRT for oligometastatic BC patients are controversial. The NRG-BR002 phase-II trial, adding MDT (surgery or SBRT) to the standard of care (SOC) for newly diagnosed oligometastatic BC at ≤ 4 extracranial sites, revealed no benefit for progression-free survival (PFS) or overall survival (OS), and has only been published in abstract form so far [14]. Similarly, no advantage was found in the CURB-phase-II trial for oligoprogressive BC with ≤5 progressive lesions [15].

Nevertheless, SBRT of BoM allows applying definitive doses to metastatic bone lesions with high precision, providing superior and more durable pain control compared to conventional radiotherapy for different histologies [16], [17], [18]. As MDT is increasingly integrated in the multidisciplinary management of oligometastatic cancer, further efforts are required to align practice patterns with existing evidence. Thus, this large European multicenter cohort study on SBRT for BoM from BC aimed to evaluate commonly applied SBRT concepts regarding oncological outcomes and safety.

## Material and methods

### Patient selection

A multicenter, retrospective cohort study on SBRT of BoM was conducted within a project of the working group on radiosurgery and stereotactic radiotherapy of the German Society for Radiation Oncology (DEGRO). Clinical data of patients, treated with bone SBRT between 2010 and 2024 at 17 European cancer centers in Germany, Austria, Switzerland, Italy, and the Czech Republic were collected and evaluated. Diagnosis of BoM was confirmed radiologically by computed tomography (CT), magnetic resonance imaging (MRI), bone scintigraphy, or positron emission tomography (PET/CT). Histological confirmation of BoM was not required. Initial BC treatment, concomitant and sequential (within 1 month after SBRT) systemic therapies during/after SBRT of BoM, further metastases (osseous/visceral), and necessary local salvage therapies after SBRT were documented. The classification and further subclassification of oligometastatic disease followed the European consensus criteria [19]. Treatment parameters of bone SBRT, including target and dose concepts and plan parameters according to the International Commission on Radiation Units and Measurements (ICRU) report 91, were documented [20], [21], [22]. Biologically effective dose (BED) was calculated using the formula BED = n × d×(1 + d/(α/β)) with n = number of fractions, d = dose per fraction, based on an α/β = 10 Gy (BED_10_) as commonly used for tumor tissue and α/β = 4 Gy (BED_4_) as a potentially more specific value for BC [23]. A minimum prescription BED_10_ of at least 44 Gy to the target volume delivered in a maximum of 10 fractions was required, thereby excluding palliative approaches.

This study has been primarily approved by the leading institutional ethics board (127/24-ek) and followed the STROBE guidelines for reporting observational studies (Supplementary Data).

### Response assessment

For response assessment, radiological findings of routinely conducted post-SBRT examinations (including CT, MRI, and PET scans) were analyzed retrospectively regarding local or distant tumor progression and fractures in the SBRT-treated region. In order to calculate freedom from local recurrence (FFLR) following SBRT, local recurrence was defined as progression in radiological imaging, and lesions without evidence of recurrence were censored at the time of the most recent imaging/radiological findings. If patients were treated with SBRT for more than one BoM, FFLR analyses were conducted independently for each lesion. OS was calculated from the end of SBRT until death from any cause. PFS was defined as the time interval from the termination of SBRT until death or the occurrence of any imaging-confirmed progression of the tumor disease. Acute (≤90 days post SBRT) and chronic (>90 days post SBRT) treatment-related adverse events were reported according to Common Terminology Criteria for Adverse Events (CTCAE) version 5.0 [24].

### Statistical analysis

All statistical analyses were conducted in Python (version 3.12.7) using in-house scripts. Data handling and preprocessing were implemented with pandas (v2.2.3) and NumPy (v2.2.1). Statistical evaluations and survival analyses were carried out using SciPy (v1.14.1) and lifelines (v0.30.0). Figures and graphical summaries were generated with Matplotlib (v3.10.0). Descriptive statistics were used to summarize patient demographics and clinical characteristics. Median values are presented together with either the 95% range or the observed value range, as appropriate. For subgroup comparisons, patient cohorts were stratified according to treatment modality and the mean BED_4_ or BED_10_ delivered to the gross tumor volume (GTV). The BED_4_/BED_10_ cutoff for dichotomization was set to 99 Gy/50 Gy in accordance to recommended dose in the practice guideline for spine SBRT (BED_10_ > 50 Gy_10_) of the European Society for Radiotherapy and Oncology (ESTRO) [11]. Time-to-event outcomes were assessed using Kaplan-Meier estimators and multivariable Cox proportional hazards regression. Kaplan-Meier curves between subgroups were compared using the log-rank test, and survival probabilities at 1 and 3 years were interpolated and reported with 95% confidence intervals (95%-CI). OS and PFS endpoints were evaluated on a patient-level, FFLR on a BoM-level. The proportional hazards assumption was verified. Multivariable models were fitted on complete cases only on preselected covariates. Continuous covariates included in the Cox models were tumor volume in ml and mean BED_10_ in the GTV in Gy. Categorical covariates were number of treated BoM (single vs. multiple), localization (non-spine vs. spine), use of systemic therapy (no vs. yes), and Her2 positive disease status. For OS, included covariates were number of treated BoM, localization, systemic therapy and Her2 positive disease status [25], [26]. For PFS, mean GTV BED_10_ was additionally included. For FFLR, GTV mean BED_10_, tumor volume and localization were included [11], [13], [27]. Her2 status was excluded owing to complete separation, which prevented model convergence. Number of treated BoM was also excluded since FFLR evaluation was BoM-specific. All results with a p-value less than 0.05 were considered significant.

## Results

Data of 109 oligometastatic BC patients, 86 (78.9%) of them with single BoM, and a total of 147 SBRT-treated BoM, were included. Detailed patient characteristics are shown in Table 1.

**Table 1:** Patient characteristics.

Variable	
*Patients (n = total)*	109
*Bone metastases (n = total)*	147
Localization in spine/non-spine	87 (59.2%)/60 (40.8%)
Single/multiple bone metastases	86 (78.9%)/23 (21.1%)
*Further metastases*	28/147 (19.0%)
Visceral/combined bone and visceral/other	6 (4.1%)/16 (10.9%)/3 (2.1%)

*Age*	
Median in years (range)	57.0 (39.2–77.2)

*Karnofsky-Index before SBRT*	
Median in % (95% range)	90% (80%-100%)
60%/70%/80%/90%/100% (n = total)	4/15/23/21/39

*Type of primary breast cancer*	
HR positive Her2 negative	109 (74.1%)
Her2 positive	29 (19.7%)
Triple negative	3 (2.0%)
No information available	6 (4.1%)

*Initial therapy primary tumor*	
*(multiple treatment modalities possible)*	
Surgery	89 (81.7%)
Radiotherapy	68 (62.4%)
Systemic Therapy	80 (73.4%)
Other	4 (3.7%)
No information available	4 (3.7%)

*Bone metastases*	
Time between initial diagnosis and bone metastasis, median (months)	52.6 (0.0–190.4)
Synchronous treatment	39 (26.5%)
Metachronous treatment	86 (58.5%)
Classification according ESTRO/EORTC [19]	
Metachronous oligoprogression	37 (25.2%)
Metachronous oligorecurrence	36 (24.5%)
Repeat oligoprogression	14 (9.5%)
Repeat oligorecurrence	9 (6.1%)
Induced oligoprogression	5 (3.4%)
Induced oligopersistence	4 (2.7%)
Induced oligorecurrence	1 (0.7%)
No information available	41 (27.9%)

*Characteristics of bone metastases*	
Radiological osteolytic	59 (40.1%)
Radiological osteoblastic	46 (31.3%)
Mixed osteolytic/osteoblastic	6 (4.1%)
Soft tissue infiltration yes/no	9 (6.0%)/114 (77.6%)
Spinal canal infiltration yes/no	0 (0.0%)/110 (74.8%)
Symptomatic before SBRT yes/no	45 (30.6%)/91 (61.9%)

*Systemic therapy during SBRT*	
Hormone therapy concomitant/sequential	63 (42.9%)/4 (2.7%)
Targeted therapy concomitant/sequential	39 (26.5%)/2 (1.4%)
Cytotoxic chemotherapy	8 (5.4%)
No systemic therapy	23 (21.1%)
No information available	1 (0.7%)

*Imaging before treatment planning:*	
Diagnostic CT	50 (34.0%)
MRI	85 (57.8%)
PET	67 (45.6%)
Scintigraphy	47 (40.0%)
No information available	1 (0.7%)
*Number of treated bone metastases per patient* Median (range)	1 (1–5)

*Treatment technique*	
C-arm-based system Photon FF/FFF	32 (21.8%)/92 (62.6%)
Robotic-based system	23 (15.6%)

Abbreviations: HR: Hormone receptor, Her: Herceptin, CT: Computed Tomography, MRI: Magnetic Resonance Imaging, PET: Positron Emission Tomography, LINAC: Linear Accelerator, FF(F): Flattening-Filter(−Free), SBRT: Stereotactic Body Radiotherapy.

The most frequently used target volume concept was the expanded GTV concept (overall: 56/147 BoM, 38.1%; spine: 16/87, 18.4%; non-spine: 40/60, 66.7%), which was defined as the BoM (GTV) supplemented only by a small treatment margin and without elective compartments. Simultaneous integrated boost (SIB) with a high dose volume covering only the BoM, and the entire vertebral body or adjacent bone tissue receiving a lower, homogeneous dose, was the second most commonly applied concept (overall: 42/147 BoM, 28.6%; spine: 35/87, 40.2%; non-spine: 7/60, 11.7%). High-dose volumes for the BoM and including the adjacent compartments (compartment concept) were administered least frequently (overall: 34/147, 23.1%; spine 32/87, 36.8%; non-spine 2/60, 3.3%).

Considering the entire cohort, SBRT was performed with a prescribed dose of median 35 Gy in median 5 fractions, this corresponds to a BED_4_ of approximately 96 Gy (corresponding BED_10_ 60 Gy). The most frequently described dose concept for spine BoM was the SIB concept with 5x 7 Gy to the high-dose volume, followed by the compartment concept using 3x 9 Gy. For non-spine BoM, the expanded GTV concept with 5x 7 Gy was most commonly applied, followed by 7x 5 Gy using the same concept. Dose-volume parameters for the different target volume concepts and treatment parameters according to ICRU 91 are presented in Table 2.

**Table 2:** Dose volume parameters according to ICRU91. Parameters presented according to the three treatment concepts.

	SIB concept	Expanded GTV concept	Compartment concept	Entire cohort
Number of treatments	42	56	34	147
Mean target volume (GTV) in ml (IQR)	4.4 (1.9–8.8)	4.9 (1.8–15.7)	10.5 (4.4–28.9)	5.4 (2.0–15.4)
Prescribed dose PTV, median in Gy (IQR)	40.0 (35.0–48.5)	35.0 (32.4–35.0)	27.5 (27.0–30.0)	35.0 (30.0–38.0)
Number of fractions, median (range)	5 (5–10)	5 (1–8)	3 (2–5)	5 (1–10)
Median PTV D_98%_ in Gy (IQR)	34.9 (32.9–38.9)	31.5 (28.4–33.1)	26.8 (24.7–28.2)	31.0 (27.0–34.3)
Median PTV D_50%_ in Gy (IQR)	41.8 (35.1–48.5)	35.0 (32.7–36.7)	31.3 (28.0–34.0)	35.0 (31.4–40.0)
Median PTV D_2%_ in Gy (IQR)	43.9 (36.4–50.6)	36.4 (35.1–39.0)	33.7 (28.9–37.6)	36.7 (33.3–42.7)
Median GTV_mean_ in Gy (IQR)	41.9 (35.8–48.5)	34.8 (33.5–35.9)	32.3 (28.6–34.8)	34.9 (32.2–41.1)
Median BED_4_ GTV_mean_ in Gy (IQR)	107.0 (96.0–127.2)	95.3 (88.9–107.5)	119.8 (91.9–137.6)	97.2 (89.9–123.7)
Median BED_10_ GTV_mean_ in Gy (IQR)	71.7 (59.8–75.8)	59.0 (52.8–64.4)	67.3 (53.4–75.3)	61.1 (54.1–73.5)
Median BED_4_ PTV D_2%_ in Gy (IQR)	114.1 (99.4–134.7)	102.5 (94.4–118.1)	144.5 (94.8–166.8)	105.1 (94.0–138.1)
Median BED_10_ PTV D_2%_ in Gy (IQR)	75.9 (61.8–80.9)	62.9 (58.3–70.8)	79.4 (54.9–86.8)	64.9 (57.2–80.5)
*Abbreviations: SIB: Simultaneous Integrated Boost, GTV: Gross Tumor Volume, PTV: Planning Target Volume, BED: Biological Effective Dose, D: Dose*				

Concomitant/sequential systemic therapy was applied in 124 of 147 lesions (84.4%), whereas 23 of 147 lesions (15.6%) received no additional systemic therapy.

The median follow-up time was 29.6 months (interquartile range: 15.0–48.0 months). During the follow-up period, 9 local recurrences were reported, and a total of 29 patients died. All local recurrences, except one with status unknown, occurred in patients with Her2 negative BC, one of those was triple negative. Analyzing the dose concepts applied, those patients received a mean BED_10_ for GTV of median 59.75 Gy (range: 51.3 Gy-79.2 Gy).

Oncological outcomes were evaluated across subgroups by dose concept based on mean BED_4_/BED_10_ for GTV.

**Fig. 1:**
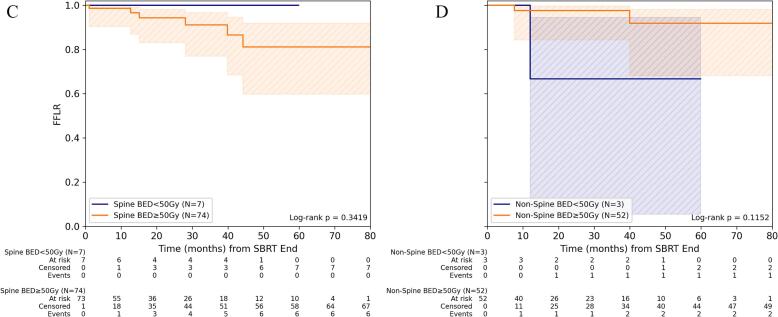

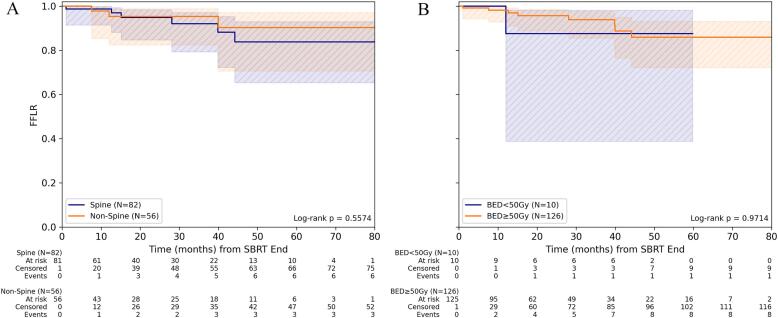
Freedom from local recurrence depending on dose concept for SBRT of bone metastasis. (A) Comparison of FFLR for spine versus non-spine lesions for the entire cohort and (B) depending on the level of mean BED_10_ (α/β = 10 Gy) in gross tumor volume (GTV). (C) Comparison of FFLR depending on the level of mean BED_10_ for spine and (D) non-spine bone metastases.

For FFLR, stratification by dose concept revealed no significant difference in patients treated with mean BED_10_ < 50 Gy/BED_4_ < 99 Gy for GTV_mean_ and those receiving a higher BED_10_ ≥ 50 Gy/BED_4_ ≥ 99 Gy. Neither for spine nor for non-spine metastases was the treatment dose associated with significant differences in FFLR. 1-/3-year FFLR for BED_10_ ≥ 50 Gy was 98.1% (95%-CI: 92.7%-99.5%)/93.9% (95%-CI: 85.5%-97.5%) and for BED_4_ ≥ 99 Gy 96.2% (95%-CI: 85.5%-99.1%)/90.0% (95%-CI: 74.6%-96.3%), respectively (Fig. 1, Supplementary Figure 1).

**Fig. 2:**
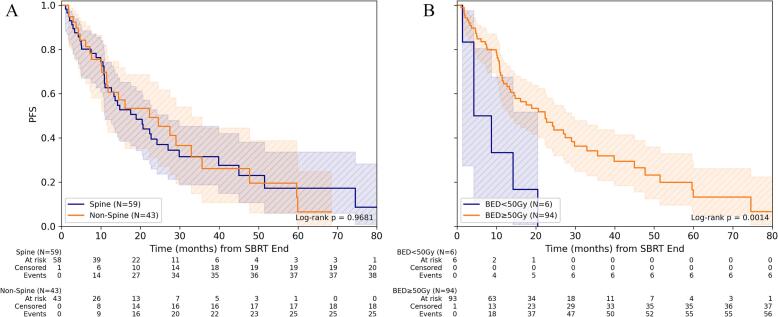

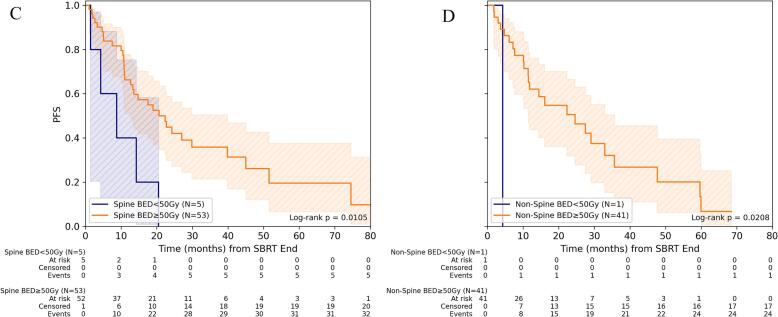
Progression-free survival depending on dose concept for SBRT of bone metastasis. (A) Comparison of PFS for spine versus non-spine lesions for the entire cohort and (B) depending on the level of mean BED_10_ (α/β = 10 Gy) in gross tumor volume (GTV). (C) Comparison of PFS depending on the level of mean BED_10_ for spine and (D) non-spine bone metastases.

For PFS, BED_10_ ≥ 50 Gy for GTV_mean_ was associated with improved PFS compared to lower BED_10_ (p < 0.002). This was evident for both spine and non-spine BoM (both p ≤ 0.02), although the subgroups here were very small and must therefore be interpreted with caution. 1-/3-year PFS for BED_10_ ≥ 50 Gy was 62.1% (95%-CI: 43.6%-76.0%)/26.7% (95%-CI: 11.0%-45.4%) for non-spine BoM and 66.2% (95%-CI: 50.8%-77.8%)/35.8% (95%-CI: 21.3%-50.4%) for spine BoM (Fig. 2, Supplementary Fig. 2).

**Fig. 3:**
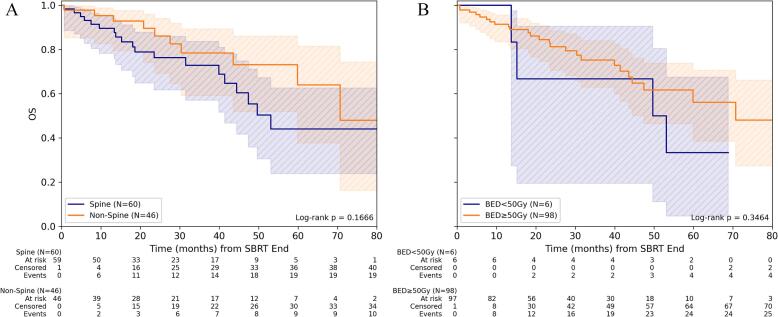

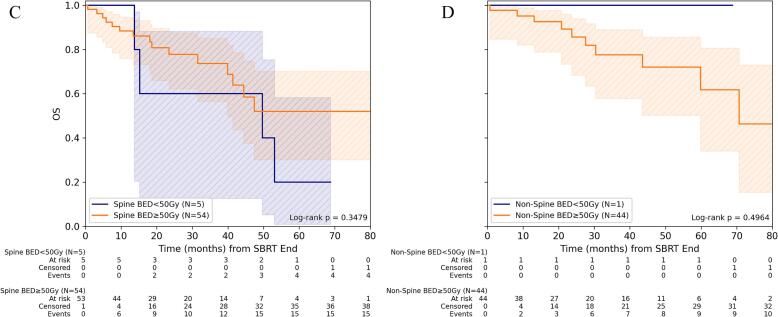
Overall survival depending on dose concept for SBRT of bone metastasis. (A) Comparison of OS for spine versus non-spine lesions for the entire cohort and (B) depending on the level of mean BED_10_ (α/β = 10 Gy) in gross tumor volume (GTV). (C) Comparison of OS depending on the level of mean BED_10_ for spine and (D) non-spine bone metastases.

For OS, no significant impact could be observed for BED_10_ ≥ 50 Gy or BED_4_ ≥ 99 Gy in GTV_mean_. 1-/3-year OS for BED_10_ ≥ 50 Gy was 91.3% (95%-CI: 83.4%-95.6%)/75.2% (95%-CI: 62.9%-84.0%) and for BED_4_ ≥ 99 Gy 89.2% (95%-CI: 76.0%-95.4%)/80.3% (95%-CI: 63.9%-89.8%), respectively (Fig. 3, Supplementary Figure 3).

In multivariable analysis, concomitant/sequential systemic therapy was associated with improved OS (HR 0.22; 95%-CI: 0.09–0.50; p < 0.001). For PFS, localization in spine was associated with less favorable PFS in multivariable analyses (HR 1.81; 95%-CI: 1.08–3.04; p = 0.02), while higher BED_10_ GTV_mean_ seemed positively associated with PFS (HR 0.96 per Gy; 95%-CI: 0.93–0.98; p < 0.001). For FFLR, none of the factors tested (GTV, BED_10_ GTV_mean_, or localization in spine/non-spine) were significant associated with better or worse FFLR. Details are presented in Table 3.

**Table 3:** Uni- and multivariable Cox proportional hazard regression analyses for overall survival, progression-free survival, and freedom from local recurrence.

Univariable	OS			PFS			FFLR		
Variable	HR (95%CI)	N	*p*	HR (95%CI)	N	*p*	HR (95%CI)	N	*p*
Age [years]	0.99 (0.96–1.02)	106	0.70	0.97 (0.95–1.00)	103	**0.03**	0.98 (0.92–1.03)	138	0.40
KPS [%]	0.95 (0.91–0.99)	105	0.08	0.99 (0.97–1.02)	102	0.46	1.05 (0.95–1.16)	137	0.32
GTV volume [ml]	1.01 (0.99–1.03)	103	0.37	1.01 (0.99–1.02)	100	0.42	1.03 (0.99–1.07)	135	0.18
BED_10_ GTV_mean_ [Gy]	−	−	−	0.97 (0.94–0.99)	101	**<0.01**	1.00 (0.94–1.06)	136	0.93
Multiple BoM	1.05 (0.76–1.43)	106	0.80	0.91 (0.71–1.16)	103	0.44	−	−	−
Spine	1.50 (0.71–3.17)	106	0.29	1.15 (0.70–1.91)	103	0.57	1.51 (0.38–6.01)	138	0.56
Systemic therapy	0.28 (0.14–0.56)	106	**<0.01**	0.76 (0.43–1.36)	103	0.36	2.18 (0.28–17.01)	138	0.45
Her2 positive	1.33 (0.54–3.29)	101	0.53	1.61 (0.95–2.76)	99	0.08	−	−	−

**Multivariable**	**OS (26 events)**			**PFS (60 events)**			**FFLR (9 events)**		

GTV volume [ml]	−	−	−	−	−	−	1.03 (0.99–1.07)	135	0.13
BED_10_ GTV_mean_ [Gy]	−	−	−	0.96 (0.93–0.98)	97	**<0.001**	0.99 (0.93–1.05)	135	0.75
Multiple BoM	1.74 (0.84–3.61)	101	0.14	0.93 (0.52–1.67)	97	0.81	−	−	−
Spine	2.20 (0.89–5.45)	101	0.08	1.81 (1.08–3.04)	97	**0.02**	1.83 (0.52–6.46)	135	0.35
Systemic therapy	0.22 (0.09–0.50)	101	**<0.001**	0.64 (0.35–1.17)	97	0.15	−	−	−
Her2 positive	0.97 (0.35–2.69)	101	0.96	1.36 (0.76–2.42)	97	0.30	−	−	−
*Abbreviations: OS: overall survival, PS: progression-free survival, FFLR: freedom from local recurrence, HR: hazard ratio, KPS: Karnofsky Performance Scale, BoM: bone metastases, CI: confidence interval, BED: Biologically Effective Dose, GTV: Gross Tumor Volume, Her: Herceptin.****Bold****: p-value < 0.05.*									

Adverse events were rare, and comprised predominantly grade 1–2 events, with fatigue being most frequent (9.5%, 14/147). Only 1.4% grade 3 adverse events (2/147, 1 acute fracture and 1 late fracture), and no grade 4 or 5 events were reported. Fracture rates related to SBRT, without tumor progression, were 0.7% for acute (1/147, expanded GTV concept) and 2.0% (3/147: 2 compartment concept, 1 expanded GTV concept) for delayed fractures (Supplementary Table 1).

## Discussion

To the best of our knowledge, this is the largest European multicenter real-world cohort analysis evaluating the outcome of patients with bone oligometastasis from BC, treated with different SBRT dose concepts, and with a particular focus on commonly used practice patterns.

We found excellent FFLR with rates exceeding 90% at 3 years and observed that higher BED_10_ for GTV_mean_ appeared to be associated with more favorable PFS, for both spine and non-spine BoM. Systemic therapy had a strong impact on the OS in the multivariable analysis and we found localization of BoM in the spine linked to worse PFS. SBRT of BoM had an excellent safety profile, with a low fracture rate of 2.7% and no grade 4 or 5 adverse events.

These findings highlight the well-known efficacy of systemic therapy in metastatic BC, and shed new light on the role of SBRT as MDT for BoM in oligometastatic BC.

In our dataset, we observed excellent 1- and 3-year FFLR of 98.1% and 93.9% for BED_10_ ≥ 50 Gy, respectively. This BED_10_ corresponds to the dose recommended in the practice guideline for spine SBRT (BED_10_ > 50 Gy_10_) of the ESTRO [11]. Due to the low number of reported local recurrences in our cohort, it was not possible to identify significant differences in local control between the different SBRT dose concepts. However, it was evident that the few local recurrences mainly occurred in patients with BoM of Her2 negative tumors.

SBRT demonstrated its value in several trials regarding excellent local control, prolonging PFS, and even partly for improving OS in the interdisciplinary treatment of oligometastases [9], [28], [29], [30], [31], [32], [33], [34]. However, the results for SBRT in oligometastatic BC have been less encouraging to date. Adding MDT, such as surgery or SBRT, to SOC for newly diagnosed oligometastatic BC at ≤4 extracranial sites failed to show a benefit for PFS or OS in the NRG-BR002 phase-II trial [14]. Bone SBRT with 1× 20 Gy (spine), 3× 10 Gy (non-spine), and 5× 7 Gy (thoracic/cervical spine) was accepted as per protocol. Incidentally, in this study, a lower progression rate was found in the regions treated with additional SBRT. In the recently published EXTEND trial, the addition of MDT (mainly SBRT) to SOC systemic therapy for oligometastatic BC (with one-fifth triple negative disease and with about one-third bone-only metastases) could not show an improved PFS, but with a very small sample size [35]. Similarly, in the CURB-phase II trial for oligoprogressive BC with ≤5 progressive lesions (with one-third of patients having triple negative BC), no advantage was found [15]. Data from corresponding phase-III studies for synchronous or metachronous oligometastatic or oligoprogressive BC are lacking. Thus, the results of the ongoing SABR-Comet-3 and −10 trials, as well as STEREO-SEIN (NCT02089100) and CORE trials (NCT02759783) will be of particular interest [36], [37].

Comparing our cohort for SBRT of BoM with available prospective trial data, we found indications for improved PFS in spine and non-spine BoM depending on the applied SBRT dose. It should be noted that our cohort included a high proportion of hormone receptor-positive (HR+) and Her2 positive disease (74.1% and 19.7%), and only a few patients with triple negative BC (2%). BoM of HR + BC and single or oligometastatic lesions have been shown to be associated with a better prognosis, compared to Her2 enriched or triple negative disease [25], [26]. In a prospective phase-II protocol for hypofractionated SBRT (≥50 Gy in 10 fractions) of oligometastatic BC, patients with solely BoM had more HR+ disease and presented with an improved OS [38], [39]. However, in another prospective phase-II trial with 2-year local control of 97% for mostly SBRT-treated oligometastasis of BC, no difference was found in terms of osseous versus visceral metastases or number of metastases (1 versus > 1 metastasis) [40].

In the absence of a significant dose-dependency for FFLR in our cohort, with nonetheless excellent local control rates, the potential additional benefit of MDT administered to systemic treatment was difficult to elucidate. The majority of patients were in excellent general condition, suggesting that this factor is unlikely to have influenced either the treatment or the decision to pursue more intensive treatment. One could assume, that some synergistic systemic effect could occur, for example, through possible immunomodulation resulting from the combination of SBRT and systemic therapy. In a small subgroup from the EXTEND trial, no significant difference in systemic T-cell activation or T-cell stimulatory cytokine concentration could be observed by the addition of MDT [35]. To date, for BC, a clear benefit for combining metastasis-directed radiotherapy with immunomodulating agents such as immune checkpoint inhibitors has not been conclusively proven [41]. Nevertheless, characterization of the tumor microenvironment (TME) of BoM from BC revealed cancer cells, bone cells, and immune cells in varying numbers and composition, apparently depending on the BC subtype [42]. Regarding the microenvironment of the primary breast tumor, high diversity has been shown in triple negative tumors, while less diversity was observed in HR+ tumors [43]. However, the TME of BC bone lesions has been described to be rather immunosuppressive, with poorer response to some immune checkpoint inhibitors [42]. Radiotherapy in combination with immunotherapies for BoM of BC has the potential to enhance treatment responses by shifting the immunosuppressive metastatic TME towards an anti-tumor immune response [44]. The possible associations between the unique microenvironment of BoM and targeted therapies, as well as possible immunomodulatory and thus synergistic effects of radiotherapy, have not yet been sufficiently analyzed and require further detailed investigation in the future.

In addition, in a recently reported randomized phase-2 trial with a high percentage of bone SBRT, even prophylactic radiation of asymptomatic, high-risk BoM of various primaries, including BC, appeared beneficial in order to reduce SRE and hospitalization [8].

Prospective studies specifically for oligometastatic BC are essential in order to verify our clinical findings and to define the subgroups that could benefit most from adding SBRT to SOC systemic therapy for oligometastatic BC.

Nevertheless, in our analysis, SBRT was an effective treatment accompanied by a favorable safety profile with a very low fracture rate. This seems remarkable, given that nearly half of the BoM in our cohort were documented as osteolytic or mixed osteolytic/osteoblastic, and it is known that patients with BC often suffer from bone loss due to hormonal anti-tumor treatment [45]. In our cohort, another third of the BoM was classified as osteoblastic lesions and hence less susceptible to fractures [46]. Broad use of hypofractionation and a younger patient age are possible reasons for a comparatively low fracture rate. A recent multivariable analysis demonstrated that increasing age, higher BED, and baseline fractures were linked to a higher risk of SBRT-related fractures [47]. In addition, in our retrospective cohort, underreporting of fractures may be a possible issue.

The current study has some limitations, primarily due to its retrospective design and use of real-world data. Due to the high number of participating centers, there was considerable heterogeneity in terms of SBRT protocols, treatment techniques, dose prescription, target or margin definition, and follow-up procedures across institutions. Additionally, the reliance on radiological reports for the assessment of local recurrence and the variability in imaging frequency could have influenced the study's outcomes. To reduce these potential limitations and in order to homogenize the data, this study focused solely on SBRT of bone oligometastasis of a specific subgroup characterized by a distinct primary diagnosis, and a predefined required minimum BED_10_ in the target volume.

Notwithstanding the mentioned limitations, our retrospective real-world cohort shows that SBRT of BoM from BC was a well-tolerated treatment with excellent local control. Further prospective studies are needed to determine the role of SBRT as a MDT for oligometastasis of BC in the context of increasingly effective systemic therapies, and to clarify its impact on long-term clinical outcomes and quality of life.

## CRediT authorship contribution statement

**Sebastian Schäfer:** Data curation, Formal analysis, Software, Visualization, Writing – original draft, Writing – review & editing. **Isabell Seiler:** Investigation, Data curation, Writing – original draft, Writing – review & editing. **Lena Kästner:** Investigation, Writing – review & editing. **Johannes Meents:** Investigation, Writing – review & editing. **Marek Slavik:** Investigation, Writing – review & editing. **Petr Burkon:** Investigation, Writing – review & editing. **Mauro Loi:** Investigation, Writing – review & editing. **Pierluigi Bonomo:** Investigation, Writing – review & editing. **Camilla von Wachter:** Investigation, Writing – review & editing. **Panagiotis Balermpas:** Investigation, Writing – review & editing. **Anna Sabrina Schunn:** Investigation, Writing – review & editing. **Sophia Drabke:** Investigation, Writing – review & editing. **Richard Holy:** Investigation, Writing – review & editing. **Kenneth Klischies:** Investigation, Writing – review & editing. **Olaf Wittenstein:** Investigation, Writing – review & editing. **Jochen Willner:** Investigation, Writing – review & editing. **Andrea Baehr:** Investigation, Writing – review & editing. **Priska Bank:** Investigation, Writing – review & editing. **Richard Partl:** Investigation, Writing – review & editing. **Thomas Mader:** Investigation, Writing – review & editing. **Bernd Frerker:** Investigation, Writing – review & editing. **Fabian Lohaus:** Investigation, Writing – review & editing. **Felix Ehret:** Investigation, Writing – review & editing. **Maike Trommer:** Investigation, Writing – review & editing. **Eleni Gkika:** Investigation, Writing – review & editing. **Alexander Rühle:** Project administration, Writing – review & editing. **Matthias Guckenberger:** Conceptualization, Writing – review & editing. **Christos Moustakis:** Project administration, Writing – review & editing. **Thomas Brunner:** Conceptualization, Writing – review & editing. **Oliver Blanck:** Conceptualization, Writing – review & editing. **Judit Boda- Heggemann:** Conceptualization, Writing – review & editing. **Nils H. Nicolay:** Conceptualization, Methodology, Project administration, Supervision, Writing – review & editing. **Franziska Nägler:** Conceptualization, Data curation, Investigation, Methodology, Project administration, Writing – original draft, Writing – review & editing.

## Ethics approval

This study was primarily reviewed and approved by the leading Ethics Committee (127/24-ek) as well as by all local Ethics Committees of the participating centers. Informed consent to participate in the study was not required. All methods used in this study were carried out in accordance with relevant guidelines and regulations.

## Funding

This research did not receive any specific grant from funding agencies in the public, commercial, or not-for-profit sectors.

## Declaration of competing interest

**LK** reports speaker fees from AstraZeneca GmbH, outside the submitted work. **PaB** reports research grants from ViewRay, Inc.; research grants, speaker fees, and travel support from Siemens Healthineers AG; speaker fees and board membership for MSD Sharp & Dohme GmbH; and board membership for Merck Healthcare Germany, all outside the submitted work. **OW** reports consulting fees from Brainlab AG and speaker fees from Novocure AG, all outside the submitted work. **FL** reports speaker fees from Regeneron Germany, Inc., outside the submitted work. **BF** reports travel grant of the German Academic Exchange Service, outside the submitted work. **FE** reports honoraria and travel support from ZAP Surgical Systems, Inc. and Accuray, Inc., and research funding from German Cancer Aid and Accuray, Inc., all outside the submitted work. **AR** reports speaker fees and research grants from Novocure AG; consulting fees from Johnson & Johnson and Need, Inc.; and speaker fees from AstraZeneca GmbH, all outside the submitted work. **MG** reports advisory board roles for Siemens Healthineers/Varian, AstraZeneca, and Johnson & Johnson, all outside the submitted work. Board membership/ ESTRO president. **TB** reports honoraria from Varian Medical Systems, Inc., Elekta AB, Brainlab AG, AstraZeneca GmbH, VisionRT, and Novocure AG, and travel fees from Onconovum, all outside the submitted work. **OB** reports a relationship with DEGRO, DGMP that includes: board membership. **JBH** reports consulting fees from EBAMed SA and speaker fees from AstraZeneca GmbH and Bristol Myers Squibb, outside the submitted work. **NHN** reports advisory board participation, speaker fees and research grants from Novocure AG; speaker fees and travel support from Merck Healthcare Germany; and speaker fees from AstraZeneca GmbH and Sun Pharmaceutical, all outside the submitted work. The other authors declare that they have no known competing financial interests or personal relationships that could have appeared to influence the work reported in this paper.

## Data Availability

The dataset generated during the current study is available from the corresponding author on reasonable request.
