# Supplementary material for: Stereotactic body radiotherapy in patients with bone oligometastases from breast cancer – results from a European multicenter cohort study
Source: Clin Transl Radiat Oncol. 2026 Mar 3;58:101142. doi: 10.1016/j.ctro.2026.101142 (PMC12992980; doi:10.1016/j.ctro.2026.101142)

Supplementary Data

Supplementary Table 1: SBRT-related acute and chronic adverse events, reported according to Common Terminology Criteria for Adverse Events (CTCAE) version 5.0 with n=number and additionally relative values in percentage of total number of lesions n=147 (100%)

**Acute toxicities**					
**CTCAE Term**	**Grade 1** n (%)	**Grade 2** n (%)	**Grade 3** n (%)	**Grade 4** n (%)	**Grade 5** n (%)
Fatigue	13 (8.84%)	1 (0.68%)	-	-	-
Pain	6 (4.08%)	1 (0.68%)	-	-	-
Esophagitis	-	1 (0.68%)	-	-	-
Coughing	1 (0.68%)	-	-	-	-
Dysphagia	1 (0.68%)	-	-	-	-
Pathological fracture	-	-	1 (0.68%)	-	-
Paresthesia	1 (0.68%)	-	-	-	-
Radiodermatitis	2 (1.36%)	-	-	-	-
Nausea	4 (2.72%)	-	-	-	-
Diarrhea	-	1 (0.68%)	-	-	-
Dizziness	-	3 (2.04%)	-	-	-
Pneumonitis	1 (0.68%)	-	-	-	-
**Chronic toxicities**					
	**Grade 1** n (%)	**Grade 2** n (%)	**Grade 3** n (%)	**Grade 4** n (%)	**Grade 5** n (%)
Pathological fracture	-	2 (1.36%)	1 (0.68%)	-	-
Pain	-	-	-	-	-
Pneumonitis	1 (0.68%)	-	-	-	-
Radiodermatitis	1 (0.68%)	-	-	-	-
Abbreviations: CTCAE: Common Terminology Criteria for Adverse Events					

Supplementary Figure 1: Freedom from local recurrence (FFLR) depending on dose concept for SBRT of bone metastasis

A) Comparison of FFLR for spine versus non-spine lesions for the entire cohort and B) depending on level of mean BED_4 (α/β=4Gy) in gross tumor volume (GTV)

C) Comparison of FFLR depending on level of mean BED_4 for spine and D) non-spine bone metastases

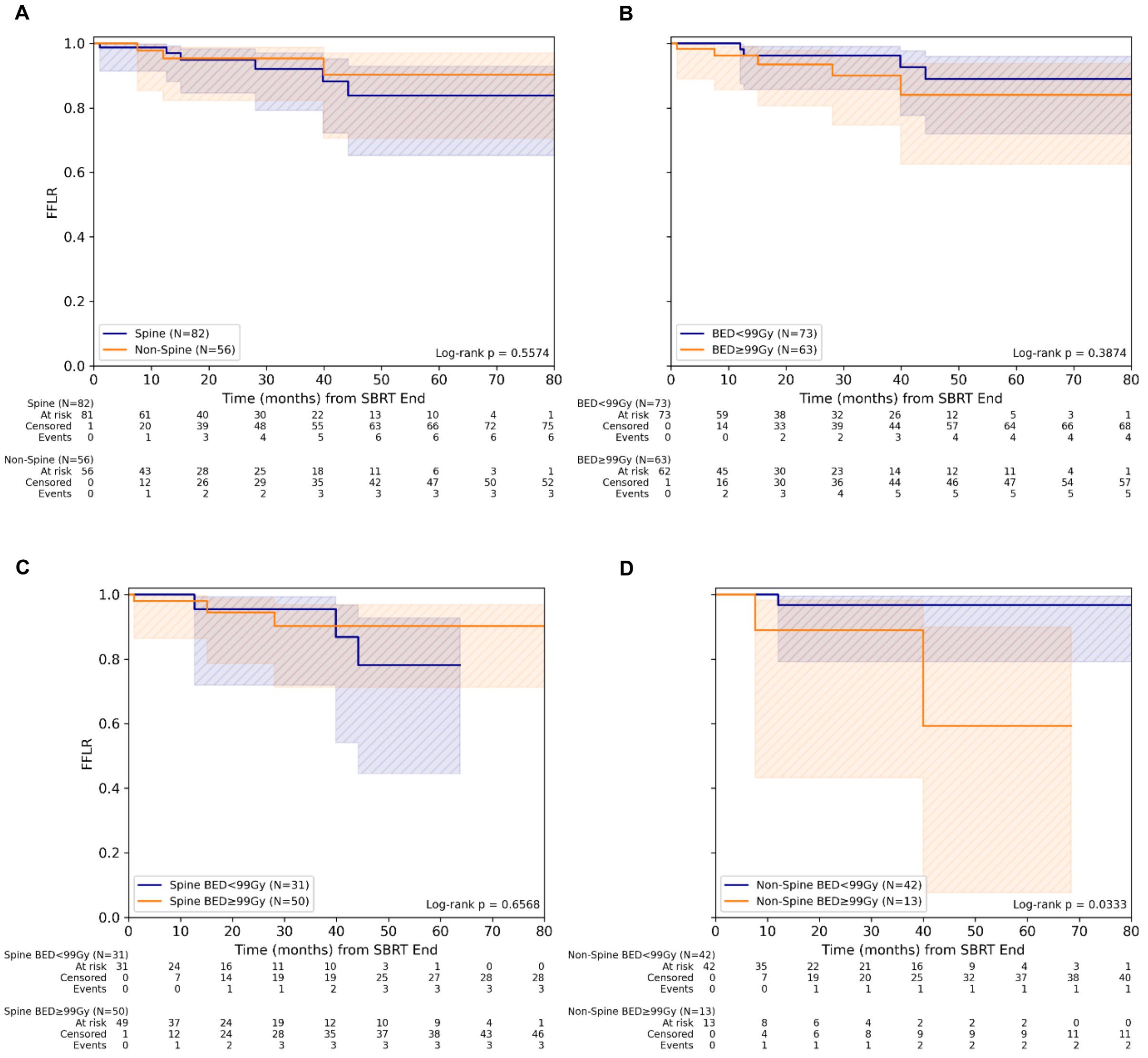

Supplementary Figure 2: Progression-free survival (PFS) depending on dose concept for SBRT of bone metastasis

A) Comparison of PFS for spine versus non-spine lesions for the entire cohort and B) depending on level of mean BED_4 (α/β=4Gy) in gross tumor volume (GTV)

C) Comparison of PFS depending on level of mean BED_4 for spine and D) non-spine bone metastases

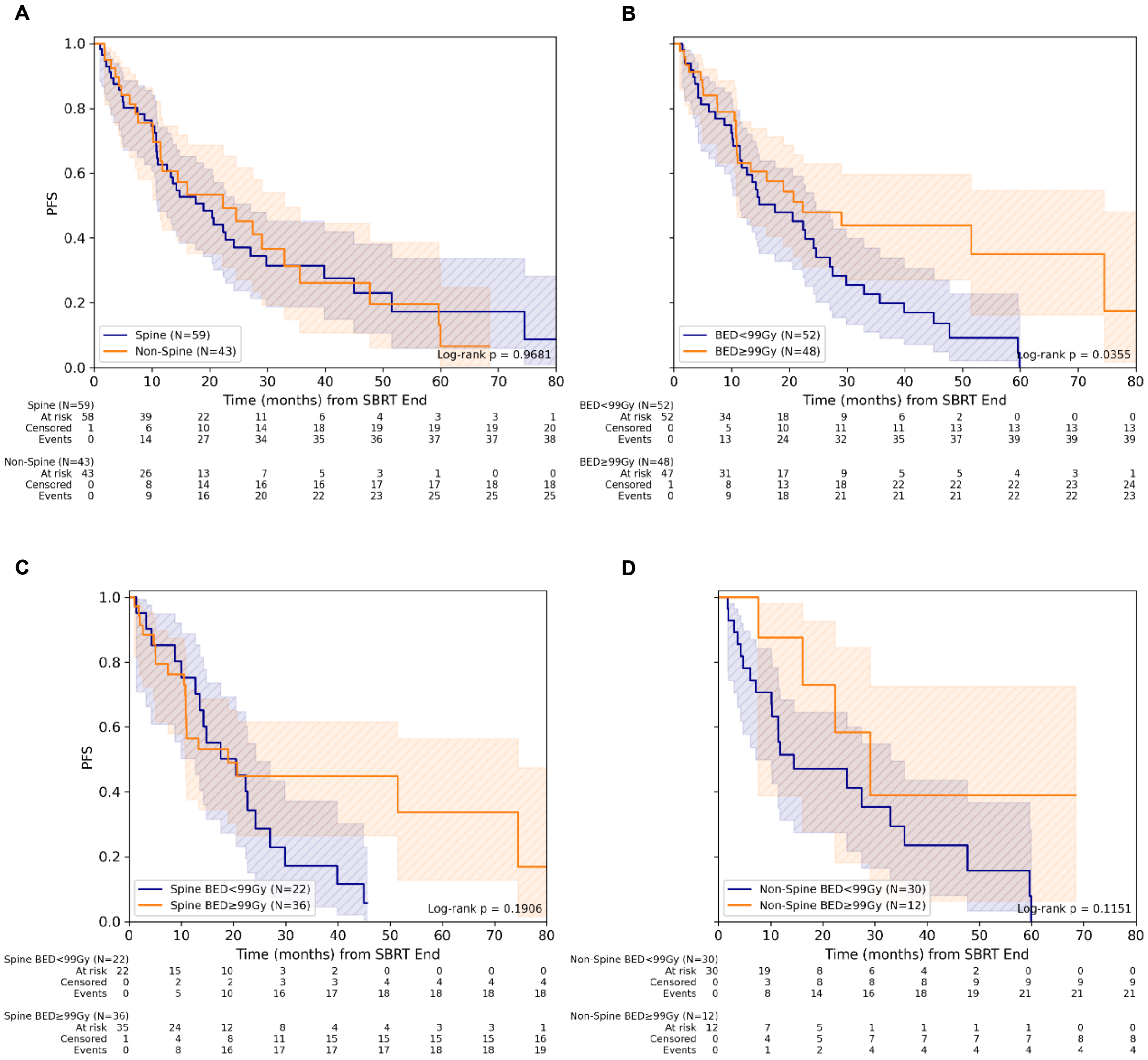

Supplementary Figure 3: Overall survival (OS) depending on dose concept for SBRT of bone metastasis

A) Comparison of OS for spine versus non-spine lesions for the entire cohort and B) depending on level of mean BED_4 (α/β=4Gy) in gross tumor volume (GTV)

C) Comparison of OS depending on level of mean BED_4 for spine and D) non-spine bone metastases

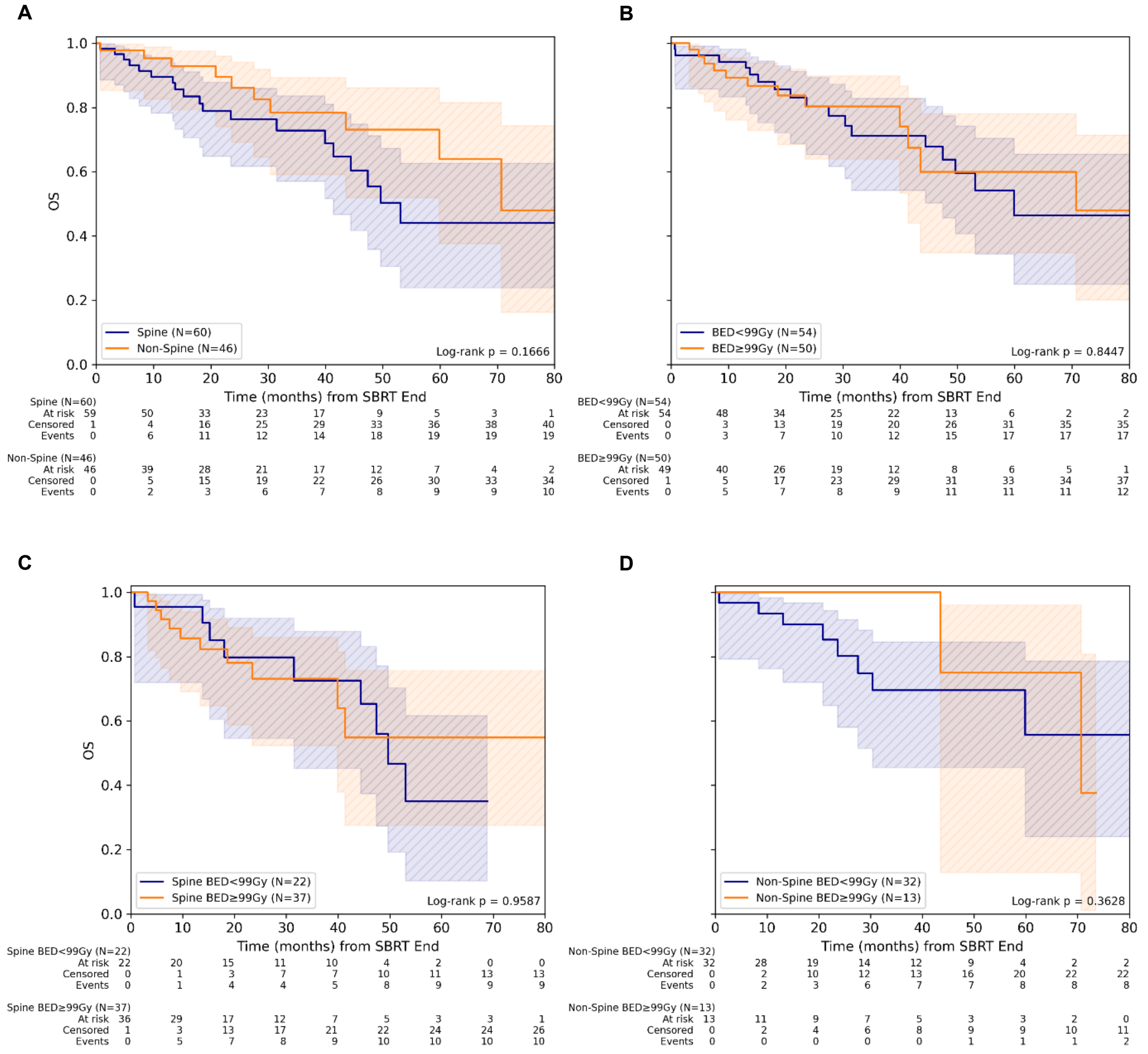

Supplement: Supplementary Data 1 [file mmc1.pdf]
